# Neurologist-focused workflows for immune effector cell-associated neurotoxicity syndrome in CAR T-cell therapy and CD3-engaging bispecific antibodies

**DOI:** 10.3389/fneur.2026.1903348

**Published:** 2026-10-14

**Authors:** Walid Alesefir, Mariam Hejazi, Basil Alashger, Abdulrahman Abodahab, Menna Abdelrazek, Karma Abouelnaga, Sara Koudeimati, Abdullah Alshibani, Lara Aljohani

**Affiliations:** 1 University of Hail College of Medicine, Hail, Saudi Arabia; 2 Almaarefa University College of Medicine, Riyadh, Saudi Arabia

**Keywords:** BCMA, bispecific antibodies, CAR T-cell therapy, electroencephalography, ICANS, neurocritical care, neurotoxicity, outpatient workflow

## Abstract

**Objective:**

To synthesize current evidence into a neurologist-focused workflow for early recognition, bedside monitoring, diagnostic triage, mitigation, and therapy-specific surveillance of immune effector cell-associated neurotoxicity syndrome (ICANS).

**Methods:**

PubMed/MEDLINE, Scopus, Web of Science Core Collection, and Google Scholar were searched in English from inception through 25 March 2026. Of 201 records, 18 duplicates were removed, 183 were screened, 157 full texts were assessed, and 26 studies were included in a narrative synthesis. The selection process was reported using applicable PRISMA 2020 elements and the narrative presentation was evaluated using SANRA.

**Results:**

Evidence supports early neurologic co-management in selected high-risk patients, serial language- and attention-sensitive bedside assessment, and clinically triggered rather than routine electroencephalography. Pilot neurocognitive data suggest that subtle decline may precede deterioration on the Immune Effector Cell-Associated Encephalopathy (ICE) score. Bispecific-antibody programs generally show lower neurotoxicity burdens than many CAR T-cell platforms but create repeated risk windows around step-up dosing and outpatient transition. Delayed B-cell maturation antigen (BCMA)-associated movement and neurocognitive syndromes remain an important safety signal, although therapeutic evidence is largely observational or case based.

**Conclusion:**

The evidence supports documenting a baseline neurologic phenotype, performing serial bedside monitoring, deploying electroencephalography and neuroimaging when results may change management, and maintaining product-specific vigilance for delayed syndromes. Tiered pathways are needed for community and resource-limited settings.

## Key points

- The strongest evidence supports disciplined serial bedside neurologic assessment, selective early electroencephalography, and product-aware escalation rather than indiscriminate testing.
- CAR T-cell ICANS and bispecific-antibody neurotoxicity overlap mechanistically but differ in timing, care setting, and recurrence logic.
- BCMA-directed CAR T-cell therapy warrants surveillance beyond the classical early ICANS window because delayed movement and neurocognitive syndromes can be clinically significant.
- Emerging neurologist-relevant tools include brief serial neurocognitive testing, selected preinfusion electroencephalography, and candidate biomarkers such as CAR-positive extracellular vesicles.

## Introduction

Immune effector therapies have transformed hematologic oncology while creating a distinct neuro-oncology workflow problem. Immune effector cell-associated neurotoxicity syndrome (ICANS) now spans adult and pediatric populations, anti-CD19 and B-cell maturation antigen (BCMA)-directed chimeric antigen receptor (CAR) T-cell products, central nervous system (CNS)-involved disease, and increasingly outpatient CD3-engaging bispecific-antibody pathways. Standardized toxicity terminology has improved communication but has not by itself created a reproducible neurologist-facing care model (1–6).

The practical questions are immediate: who merits pretreatment neurologic assessment, how frequently bedside testing should be repeated, when electroencephalography (EEG), magnetic resonance imaging (MRI), or cerebrospinal fluid (CSF) analysis may change care, and how long delayed syndromes should remain in the differential. These questions become more important as bispecific antibodies move into outpatient and community settings, where observation and transfer systems may be as consequential as drug-specific risk (2–6).

A recent scoping review by Alqaisi et al. (7) illustrates the expanding neurologic scope of CAR T-cell therapy across hematologic malignancies, CNS tumors, and autoimmune neurologic disease and emphasizes that toxicity burden varies by disease and therapeutic context. The present review focuses specifically on neurologist-facing ICANS workflows: baseline assessment, serial monitoring, trigger-based testing, therapy-specific timing, mitigation, and post-acute surveillance.

## Methods

### Review design and reporting

This structured mini-review examined neurologist-facing strategies for recognizing, monitoring, evaluating, and mitigating neurotoxicity associated with CAR T-cell therapy and CD3-engaging bispecific antibodies. The search and selection process was reported using applicable elements of the Preferred Reporting Items for Systematic Reviews and Meta-Analyses 2020 statement (PRISMA 2020). Narrative presentation was evaluated using the Scale for the Assessment of Narrative Review Articles (SANRA).

### Information sources and search period

PubMed/MEDLINE, Scopus, Web of Science Core Collection, and Google Scholar were searched from database inception through 25 March 2026. Searches were conducted between 7 and 25 March 2026. Sixty Google Scholar records were selected for screening.

### Search strategy

The exact PubMed/MEDLINE strategy was: (“immune effector cell-associated neurotoxicity syndrome” OR ICANS OR neurotoxicity) AND (“CAR T-cell” OR “chimeric antigen receptor T-cell” OR “CD3-engaging bispecific antibody” OR “bispecific antibody” OR teclistamab OR elranatamab OR mosunetuzumab OR glofitamab OR epcoritamab) AND (neurolog* OR EEG OR electroencephalograph* OR MRI OR neuroimag* OR cognit* OR language OR attention OR seizure* OR monitoring OR management OR treatment OR prophylaxis OR mitigation OR movement OR parkinsonism). Syntax was adapted for other databases. Only English-language peer-reviewed publications were eligible. Conference abstracts, editorials, commentaries, preprints, and other non-peer-reviewed publications were excluded.

### Eligibility criteria

**Table:** 

Dimension	Included	Excluded
Population	Recipients of CAR T-cell or CD3-engaging bispecific-antibody therapy	Other therapies or populations without relevant immune effector exposure
Intervention/exposure	CAR T-cell therapy; CD3-engaging bispecific antibodies	Preclinical exposure without directly applicable clinical findings
Comparator	Not required	Not applicable
Outcomes	ICANS recognition/grading; serial monitoring; EEG/imaging; timing; neurologic management/mitigation; outpatient workflows; delayed BCMA syndromes	No ICANS or extractable clinically relevant neurologic outcome
Study type	Consensus guidance, reviews, meta-analyses, trials, observational studies, real-world cohorts, pilots, clinically informative case reports	Conference abstracts, editorials, commentaries, preprints, non-peer-reviewed reports, overlapping reports without additional findings
Language	English	Non-English

### Study selection and data extraction

Records were consolidated and duplicates identified using DOI, PMID, title, authors, year, and manual comparison. Primary title/abstract screening, full-text assessment, and data extraction were performed by BKA. MZH reviewed the final included studies, verified principal extracted findings, and participated in resolving uncertain decisions. Disagreements were resolved by consensus, with consultation of the lead author when required. Screening therefore used one primary reviewer with secondary verification rather than two fully independent reviewers.

The searches identified 201 records: PubMed/MEDLINE (*n* = 80), Google Scholar (*n* = 60), Scopus (*n* = 25), Web of Science (*n* = 20), and publisher/reference-list searching (*n* = 16). After 18 duplicates were removed, 183 records were screened and 26 were excluded at title/abstract level. Of 157 full-text reports assessed, 131 were excluded: no relevant neurologic outcome (*n* = 36), wrong population/intervention (*n* = 24), no clinically useful workflow information (*n* = 22), preclinical/predominantly mechanistic evidence (*n* = 17), insufficient clinical detail (*n* = 14), ineligible type or unavailable full text (*n* = 10), and overlapping cohort without additional findings (*n* = 8). Twenty-six studies were included (Figure 1).

**Figure 1:**
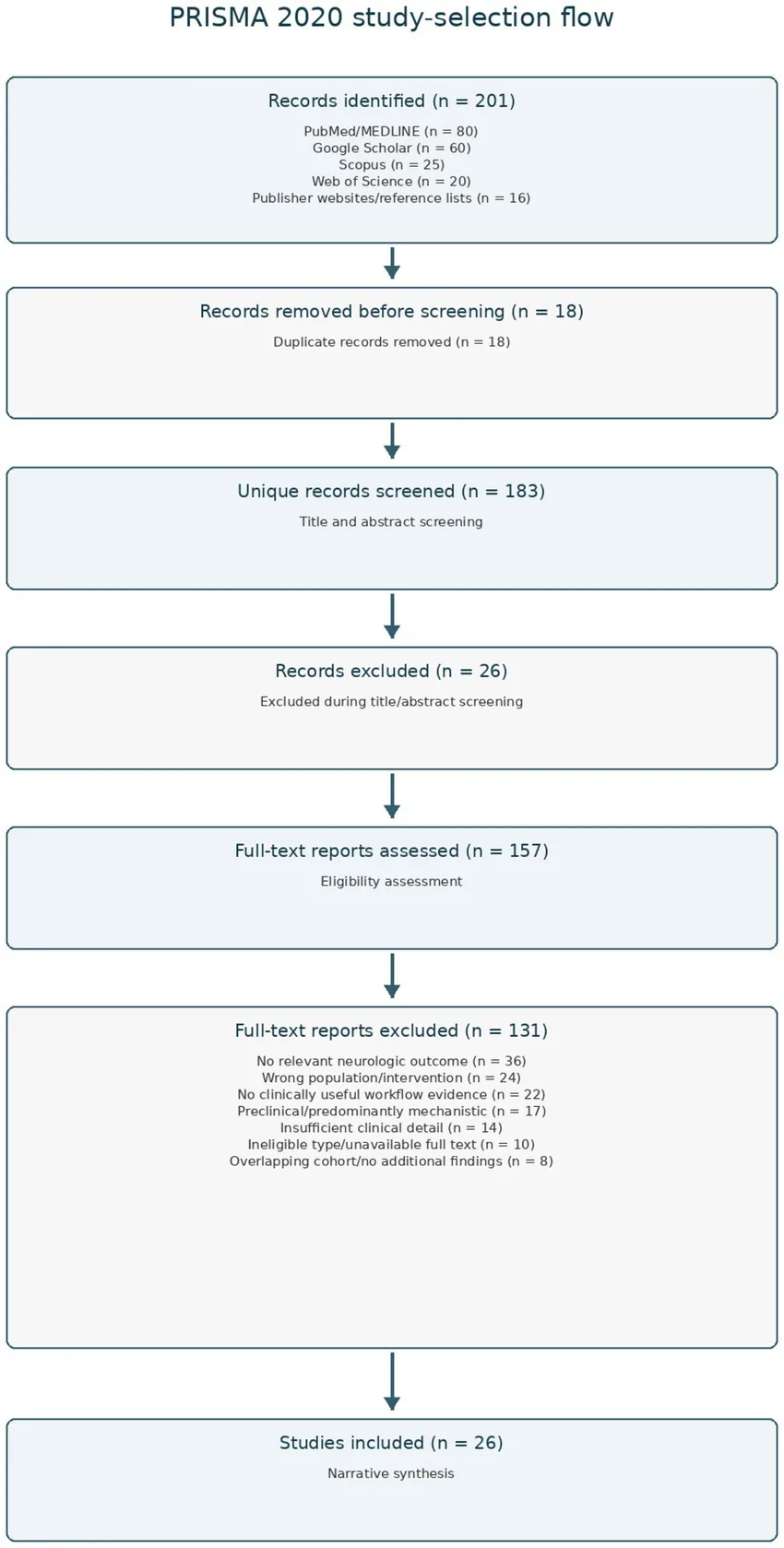
PRISMA 2020 flow diagram of identification, screening, eligibility assessment, and inclusion.

For each study, design, population, therapy, neurologic phenotype, timing, diagnostic or monitoring approach, principal findings, limitations, and workflow relevance were extracted. Clinical and methodological heterogeneity precluded quantitative pooling; evidence was grouped by clinical decision domain and synthesized narratively.

### Quality appraisal and evidence weighting

Because the included evidence comprised heterogeneous guidance, reviews, trials, observational studies, pilots, and case reports, recommendations were stratified by source design and directness rather than assigned a single pooled certainty estimate. Meta-analyses, consensus guidance, and comparative trials were treated as higher-level contextual evidence; observational cohorts and real-world studies as moderate or limited evidence depending on design and size; pilot studies as preliminary; and case reports as safety signals or hypothesis-generating evidence only. Appendix reports design-specific limitations and narrative evidence strength. This approach does not substitute for a full outcome-level GRADE assessment and is acknowledged as a limitation.

### Relationship to previous work

The source library was created specifically for this manuscript and was not derived from another manuscript, thesis, registered protocol, conference abstract, or overlapping publication project. No text, figures, tables, or analyses from this review have been published previously.

### Evidence-based overview

The included evidence comprises consensus guidance, reviews, meta-analyses, trials, prospective pilots, observational cohorts, real-world series, biomarker studies, and case reports. It is strongest for standardized grading and broad workflow principles but weaker for the comparative effectiveness of specific monitoring intensities, prophylactic antiseizure therapy, biomarker-guided surveillance, and rescue treatment of delayed movement syndromes.

**Table:** 

Evidence domain	Included studies	Interpretive weight
Direct neurologic workflow/management	10	Consensus and implementation guidance; useful operational evidence but limited comparative testing
Neurodiagnostic/biomarker evidence	8	Mostly observational or pilot evidence; supports selective testing and future validation
Direct neurotoxicity/ICANS evidence	6	Mixed meta-analytic, cohort, and case-level evidence
Therapy-specific safety context	2	Clinical-trial context; not designed primarily to compare neurologic workflows

### Why workflows differ by therapy class

ICANS is better conceptualized as a syndrome family than a fixed entity. The ASTCT/ASBMT framework standardized toxicity language and remains foundational (1), but identical workflows cannot be assumed across platforms. CAR T-cell programs concentrate risk early after infusion while also producing selected delayed syndromes, especially after BCMA-directed therapy. Bispecific antibodies often produce lower-grade neurotoxicity on average, yet step-up dosing and outpatient administration create repeated observation, communication, and transfer challenges (2–6) (see Figure 2).

**Figure 2:**
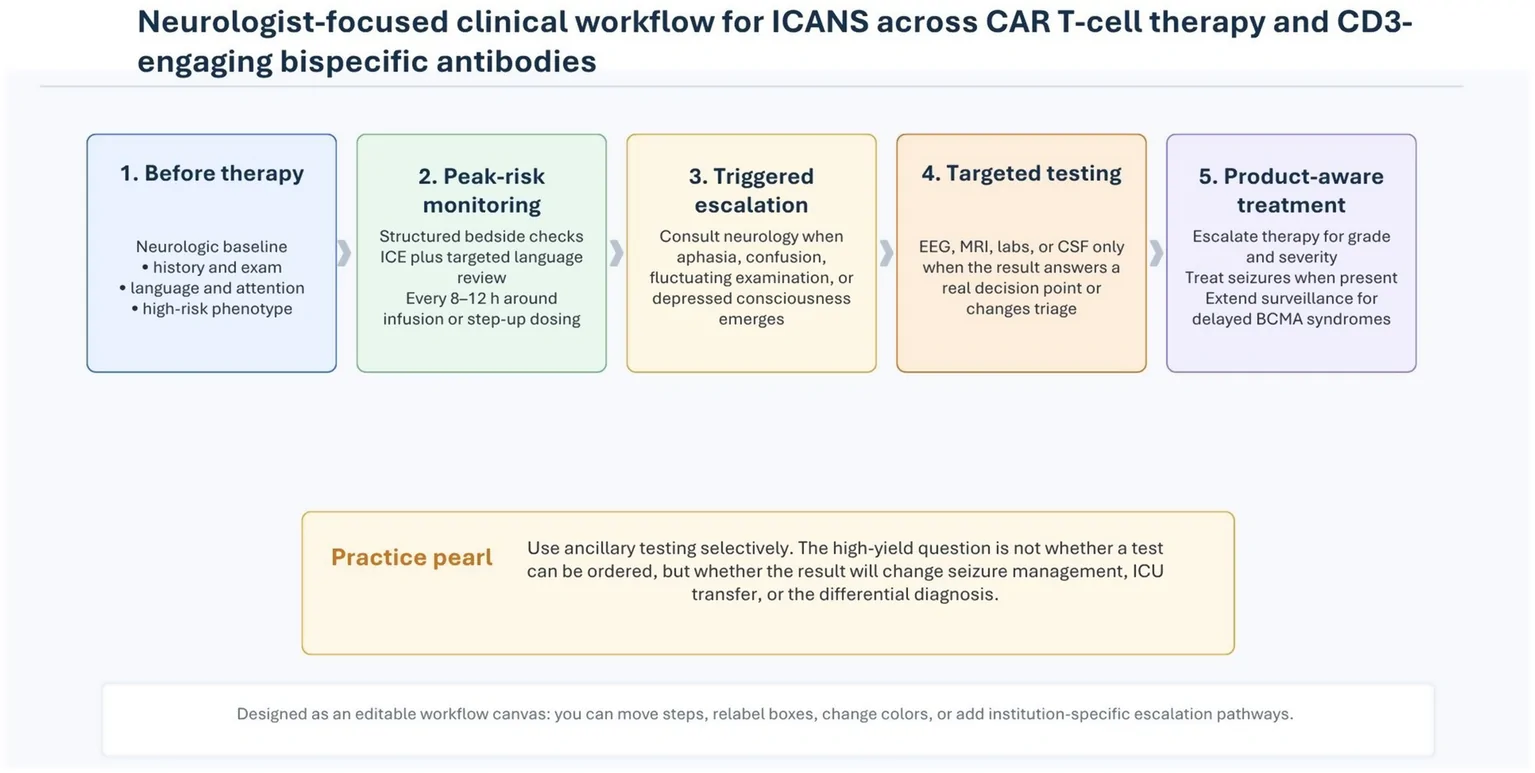
Neurologist-focused clinical workflow across CAR T-cell and CD3-engaging bispecific-antibody therapy.

### Baseline assessment and serial monitoring

Pretreatment assessment should document prior stroke or seizure, CNS disease, baseline language and cognition, gait or movement abnormalities, and medications that may confound the examination. The purpose is not exhaustive testing of every patient but recognition of pre-existing deficits and identification of patients in whom subtle change would be difficult to interpret.

During the highest-risk interval, scheduled bedside assessment should combine the Immune Effector Cell-Associated Encephalopathy (ICE) score with targeted attention to language, writing, attention, fluency, consciousness, and new abnormal movements. In the 2025 CART-NS pilot, brief serial neurocognitive testing detected subtle change and early Stroop decline predicted subsequent ICANS with an area under the receiver operating characteristic curve of 0.857 (8). This is preliminary pilot evidence and does not establish one mandatory universal battery.

### EEG, imaging, and CSF triage

**Table:** 

Test	Best triggers	Clinical role	Evidence qualification
EEG	Fluctuating examination, reduced responsiveness, suspected nonconvulsive seizure, myoclonus, grade ≥2 encephalopathy	Distinguish seizure from diffuse encephalopathy and support severity assessment	Observational EEG series; selective early use supported, routine universal testing unproven
Selected preinfusion EEG	Seizure history, CNS disease, baseline cognitive abnormality, anticipated diagnostic ambiguity	Risk context and baseline comparison	Single observational adult study; association does not prove clinical benefit
MRI/CT	Focal deficit, prolonged or refractory course, concern for stroke, hemorrhage, infection, or edema	Exclude structural alternatives	Adjunctive diagnostic role; routine imaging in mild ICANS unsupported
CSF	Atypical or refractory course with concern for infection, malignant CNS involvement, or competing inflammation	Narrow differential diagnosis	Selective use based mainly on guidance and clinical reasoning

In a 29-patient adult ICANS EEG series, diffuse slowing predominated and electrographic seizures were not observed after patients had entered established treatment pathways (9). A separate 56-patient study associated abnormal preinfusion EEG with severe ICANS (50% versus 17%), with ICANS occurring at a median of 6 days (10). These observational findings favor early question-driven EEG but do not justify routine baseline EEG for every patient.

### Risk factors, biomarkers, and early warning signals

Patient vulnerability, therapy class, tumor and inflammatory burden, and dynamic biological signals may modify risk. Candidate signals include cytokine changes measured in the CART-NS pilot, CAR-positive extracellular vesicles in CD19-directed therapy, and fludarabine exposure in idecabtagene vicleucel cohorts (8, 11, 12). These findings remain investigational and should not replace serial clinical assessment.

### Therapy-specific patterns

**Table:** 

Therapy context	Main neurologic pattern	Operational implication	Evidence level
Anti-CD19 CAR T-cell	Early encephalopathy, aphasia, seizure concern, EEG slowing	Structured monitoring and low threshold for clinically triggered EEG	Consensus, meta-analysis, trial, and observational evidence
CD3-engaging bispecific antibodies	Generally lower-grade events but repeated risk around step-up dosing	Observation, explicit escalation pathways, and continuity across settings	Meta-analysis, implementation guidance, and real-world cohorts
BCMA-directed CAR T-cell	Early ICANS plus delayed movement and neurocognitive syndromes	Extended symptom-specific follow-up after apparent recovery	Cohort safety signal plus case-report-level evidence for rare delayed phenotypes/rescue

A meta-analysis of 32 bispecific-antibody trials involving 2,192 patients with non-Hodgkin lymphoma quantified aggregate safety signals, while implementation guidance translated risk into outpatient observation and transfer requirements (4, 6). In a 119-patient real-world prophylaxis cohort, tocilizumab before the first bispecific dose was associated with observed cytokine release syndrome (CRS) and ICANS rates of 10.1 and 5.9%, respectively, without an apparent efficacy penalty (13). The nonrandomized design precludes causal conclusions or universal prophylaxis recommendations. Reports of refractory teclistamab neurotoxicity also caution against equating lower average risk with absence of severe events (14–16).

Delayed BCMA-associated movement and neurocognitive syndromes extend surveillance beyond the classical early window (17–20). Cohort data establish an important program-level safety signal, whereas individual delayed phenotypes and proposed rescue treatments remain supported largely by case reports. These reports support vigilance but do not establish incidence, comparative risk, or treatment efficacy (see Figure 3).

**Figure 3:**
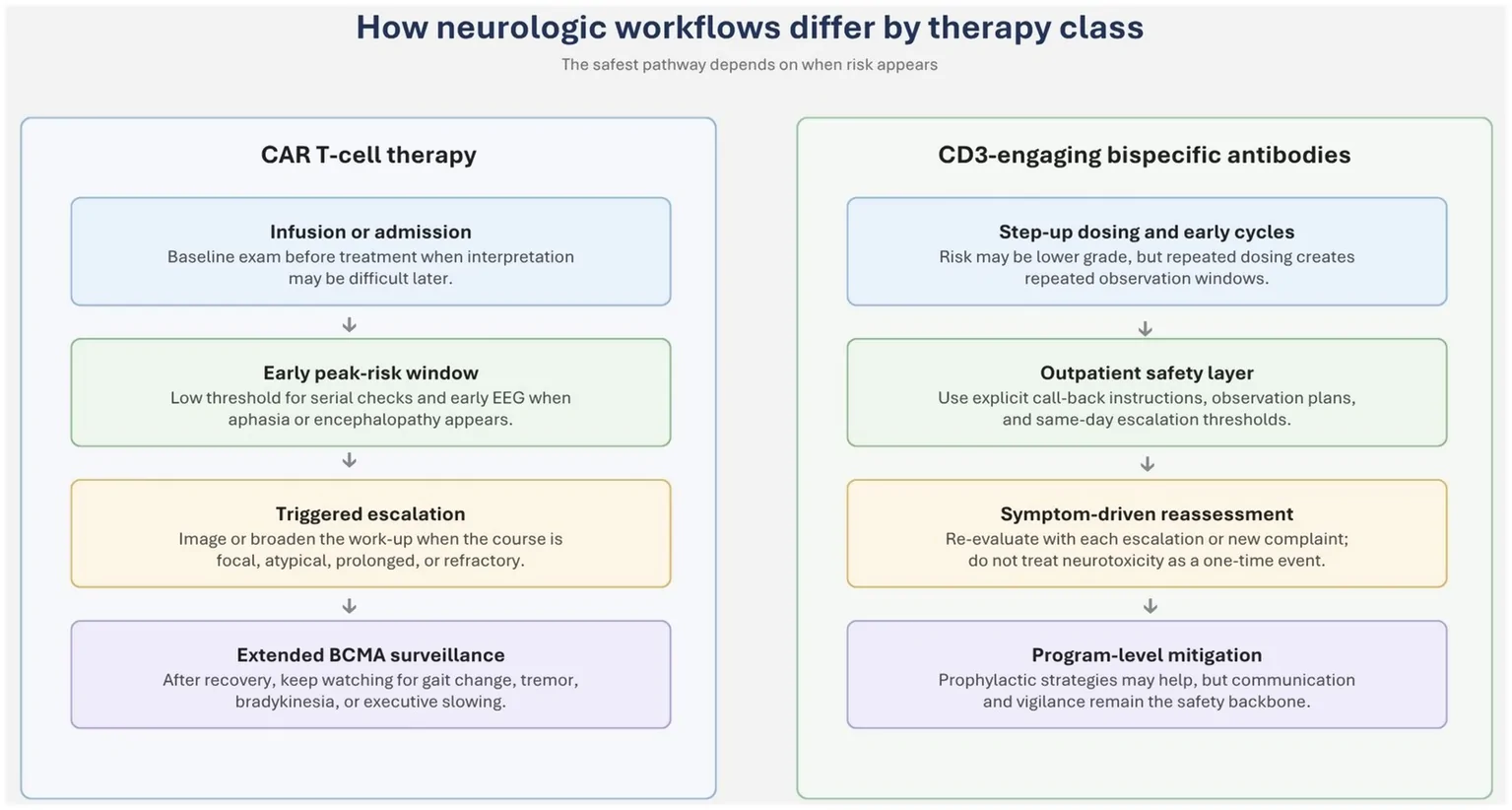
Conceptual timing differences that shape neurologic surveillance. Timing is product- and patient dependent; the figure is not a fixed dosing calendar.

### Product-specific patterns: CAR T-cell versus bispecific programs

**Table:** 

Therapy context	Main neurologic pattern	Operational implication	Key included evidence
Anti-CD19 CAR T-cell therapy	Classical early ICANS: encephalopathy, aphasia, seizure concern, EEG slowing	Structured inpatient monitoring remains central; low threshold for early EEG when exam fluctuates.	Consensus grading, EEG studies, pediatric neurotoxicity cohorts, CNS lymphoma meta-analysis, and product-specific safety context (1, 8–10, 21–26)
CD3-engaging bispecific antibodies	Usually lower-grade neurotoxicity than CAR T, but recurrent exposure and step-up dosing create repeated risk windows.	Safe use depends on observation systems, explicit escalation pathways, and—where adopted—mitigation strategies such as prophylactic tocilizumab.	Meta-analyses, outpatient roadmap, prophylaxis cohort, and real-world teclistamab studies (4–6, 13–16, 27)
BCMA-directed CAR T-cell therapy	Early ICANS plus delayed movement/neurocognitive syndromes, including Parkinsonism-like presentations	Extend surveillance beyond initial recovery; specifically ask about gait, bradykinesia, executive decline, and late confusion.	CARTITUDE movement/neurocognitive reports, delayed case literature, rescue reports, and ide-cel toxicity analyses (12, 17–20)

### Proactive mitigation and areas of uncertainty

**Table:** 

Mitigation domain	Most defensible current use	Practical caveat	Supporting included studies
Early steroid escalation	When neurologic decline is clinically meaningful, and alternative diagnoses have been addressed	Best used as part of a workflow that includes neurology input, severity grading, and seizure exclusion when appropriate	Consensus and review-level guidance (1–3)
Antiseizure therapy	Treat witnessed or electrographic seizures; consider a lower threshold in clearly high-risk severe cases.	The current included evidence does not support routine prophylaxis for all patients	EEG series and review-level guidance (2, 3, 9, 10, 25)
Tocilizumab prophylaxis in bispecific programs	Reasonable program-level strategy where step-up dosing logistics and prior institutional experience support it	Needs integration with local pathways and continued neurologic vigilance because low event rates do not equal zero risk	Real-world 119-patient cohort with low CRS and ICANS rates (13)
ICU transfer/continuous EEG	Severe encephalopathy, coma, recurrent seizure concern, hemodynamic instability, or multi-organ toxicity	Triggered escalation is more efficient than blanket ICU use	Consensus workflow literature and adult EEG data (1, 9)
Late-syndrome surveillance and rescue	Movement disorder or executive decline after BCMA-directed therapy should prompt renewed neurologic evaluation; rescue strategies may differ from classical early ICANS	Delayed syndromes may not respond like typical dopaminergic disease; case literature supports alternative immunomodulatory approaches in selected settings.	Cilta-cel MNT analyses and delayed rescue literature (17–20)

Clinically meaningful deterioration should prompt rapid severity assessment, evaluation for competing diagnoses, and timely treatment according to local immune effector toxicity protocols. Antiseizure medication is indicated for witnessed or electrographic seizures, but evidence does not support routine prophylaxis for every recipient. The optimal threshold for corticosteroids in early or evolving ICANS, the value of universal antiseizure prophylaxis, the definition of “high risk” for baseline EEG, and the role of tocilizumab prophylaxis in bispecific programs remain contested or insufficiently tested. Recommendations should therefore distinguish consensus-driven management of established toxicity from selective strategies supported mainly by observational data.

A case report described improvement with ruxolitinib in ciltacabtagene autoleucel-associated Parkinsonism (20). This isolated observation is hypothesis-generating and does not establish efficacy (see Box 1).

**BOX 1 When to call neurology urgently**

- Any new aphasia, agraphia, marked inattentiveness, or rapidly fluctuating mental status after immune effector therapy.
- Reduced responsiveness, concern for nonconvulsive seizure, new myoclonus, or paroxysmal events that cannot be confidently classified at the bedside.
- Delayed gait disorder, bradykinesia, tremor, executive decline, or behavioral change after BCMA-directed CAR T-cell therapy, even when the early post-infusion period has already passed.
- Any severe or atypical course in which MRI, CSF sampling, ICU transfer, or continuous EEG is being considered.

### Implementation in community and resource-limited settings

Most supporting evidence originates from tertiary or academic centers with ready access to neurology, EEG, MRI, intensive care, and cellular-therapy teams. A tiered approach may improve transferability. At minimum, programs should document a brief baseline neurologic examination, identify pre-existing language or cognitive deficits, use a standardized bedside score during defined risk periods, educate patients and caregivers about aphasia, confusion, reduced responsiveness, abnormal movements, and delayed gait or cognitive changes, and establish explicit transfer pathways. When immediate EEG is unavailable, urgent transfer should be considered for unexplained reduced responsiveness, fluctuating encephalopathy, or suspected seizure. Preinfusion EEG and formal serial neurocognitive batteries should be reserved for selected patients when resources permit rather than treated as universal prerequisites. Tele-neurology, caregiver-assisted observation, written escalation instructions, and scheduled telephone follow-up are pragmatic alternatives requiring prospective validation.

### Pediatric considerations

Pediatric and young-adult studies support serial neurologic assessment and demonstrate correlations between EEG abnormalities and neurotoxicity severity (23, 25), but adult workflows cannot be transferred without qualification. ICE is primarily an adult tool; age-appropriate assessment, including the Cornell Assessment of Pediatric Delirium where applicable, may be needed in younger children. Pediatric monitoring and escalation should be individualized with pediatric neurology and critical-care input. Current evidence is insufficient to define a validated pediatric-specific workflow.

### Patient- and caregiver-reported outcomes

Patient-reported neurologic symptoms, caregiver observations, health-related quality of life, and caregiver burden are underrepresented in the included literature. Caregivers may recognize language, behavioral, gait, or executive changes before episodic clinical assessments, especially after discharge. Future studies should combine clinician-administered toxicity grading with validated patient- and caregiver-reported measures.

### Limitations

This review has several limitations. Screening was performed by one primary reviewer with secondary verification rather than two independent reviewers, increasing the possibility of selection error. Restriction to English-language peer-reviewed literature. The heterogeneous designs precluded quantitative synthesis and a unified risk-of-bias instrument. Many workflow recommendations derive from tertiary centers, limiting direct generalizability. Evidence for delayed BCMA syndromes includes small cohorts and case reports, and proposed rescue therapies remain hypothesis-generating. Conflicting practices concerning early corticosteroids, antiseizure prophylaxis, and baseline EEG have not been resolved by comparative trials. Pediatric workflows, patient-reported outcomes, quality of life, and caregiver burden remain incompletely studied.

## Conclusion

The available evidence supports documenting a baseline neurologic phenotype, performing serial language- and attention-sensitive bedside assessment, and using EEG, imaging, and CSF analysis selectively when results may alter management or exclude important alternatives. Therapy-aware pathways should distinguish the concentrated early risk of CAR T-cell therapy, repeated observation windows around bispecific-antibody step-up dosing, and delayed movement or neurocognitive safety signals after BCMA-directed therapy. Evidence for universal preinfusion EEG, broad antiseizure prophylaxis, biomarker-guided care, and specific rescue therapy for delayed syndromes remains insufficient. Tiered pathways adapted to local resources and stronger prospective evidence are needed.

## Appendix

**Table A1:** Evidence and narrative quality appraisal.

No.	Study	Design	Principal limitation	Narrative evidence strength
1	Lee et al.	Consensus guidance	Foundational standardized grading; consensus-based rather than comparative effectiveness evidence	Moderate–high contextual authority
2	Fatahichegeni et al.	Review	Broad recent synthesis; dependent on included literature and review methods	Contextual review evidence
3	Ruff et al.	Focused review	Neurologist-oriented practical synthesis; not comparative	Contextual review evidence
4	Garfall et al.	Implementation review	Operationally useful outpatient roadmap; center/resource dependent	Consensus/implementation evidence
5	Bayly-McCredie et al.	Systematic review	Aggregates clinical trials; heterogeneity and trial-selection limitations	Systematic-review level
6	Fonseca et al.	Meta-analysis	Large aggregate safety context; trial heterogeneity and indirect workflow evidence	Meta-analysis level
7	Cook et al.	Meta-analysis	CNS lymphoma safety context; small heterogeneous cohorts	Meta-analysis with imprecision
8	He et al.	Clinical cohort/trial	Direct CNS-involved population; product- and setting-specific	Limited–moderate clinical evidence
9	Shalabi et al.	Prospective pediatric/AYA study	Detailed phenotype; limited generalizability to adults/products	Preliminary prospective evidence
10	Guo et al.	Pharmacovigilance study	Large real-world signal detection; reporting and denominator bias	Signal-generating observational evidence
11	Suresh et al.	Prospective pilot	Sensitive serial testing and predictive signal; small pilot requiring validation	Preliminary pilot evidence
12	Wesley et al.	Observational EEG series	Direct EEG characterization; selected treated population and small sample	Limited observational evidence
13	Gust et al.	Pediatric EEG study	Prospective physiologic correlations; pediatric/product-specific	Limited prospective evidence
14	Hernani et al.	Observational EEG study	Baseline association with severity; nonrandomized and modest sample	Limited observational evidence
15	Storci et al.	Biomarker study	Novel predictive candidate; requires external clinical validation	Exploratory biomarker evidence
16	Sweiss et al.	Real-world cohort	Exposure–toxicity association; confounding and product specificity	Moderate observational evidence
17	Abramson et al.	Phase 3 trial	Robust product safety context; workflow not primary endpoint	High product-context evidence
18	D’Souza et al.	Phase 1 trial	Direct early safety data; small, noncomparative dose-escalation study	Early-phase evidence
19	Mohan et al.	Real-world cohort	Multicenter practical safety context; retrospective confounding	Moderate observational evidence
20	Dima et al.	Real-world cohort	Academic-center experience; selection and retrospective limitations	Limited–moderate observational evidence
21	Lutfi et al.	Case-based report	Highlights rare severe refractory toxicity; cannot estimate incidence	Case-level safety signal
22	Kowalski et al.	Real-world prophylaxis cohort	Operationally relevant association; no randomized control	Limited–moderate observational evidence
23	Cohen et al.	CARTITUDE cohort analysis	Program-level delayed toxicity signal; product-specific	Moderate observational safety evidence
24	Schroeder et al.	Case report	Rare delayed phenotype; no generalizable frequency or causality	Case-report-level evidence
25	Khoudari et al.	Case report	Severe delayed safety signal; no incidence inference	Case-report-level evidence
26	Wirk and Lim	Case report	Therapeutically informative single observation; efficacy unproven	Case-report-level evidence only
27	Alqaisi et al.	Scoping review	Broad neurologic disease context; complementary rather than part of original 26	Scoping-review context
